# Daily QA in proton therapy using a single commercially available detector

**DOI:** 10.1120/jacmp.v15i6.5005

**Published:** 2014-11-08

**Authors:** Jamil Lambert, Christian Bäumer, Benjamin Koska, Xiaoning Ding

**Affiliations:** ^1^ Westdeutsches Protonentherapiezentrum Essen (WPE) GmbH Essen Germany

**Keywords:** PBS, pencil beam scanning, US, uniform scanning, routine QA

## Abstract

We present here a novel method for using a single device in the daily quality assurance (QA) of pencil beam scanning (PBS) proton beams and an improved method for uniform scanning (US). The device can be used to measure the spot position, spot sigma, range, output, collinearity of the X‐ray system and proton beam, and to QA the first scatterers and a number of other imaging and mechanical checks. We have performed the daily QA according to this procedure for more than six months in both a PBS gantry and a US gantry. All of the tests were found to be sensitive and accurate enough to determine if the property being tested is within the tolerance. The output has remained within the ±2% tolerance, with the majority of measurements within ±1%, and the range was within ±0.5mm. The collinearity of the proton beam in both gantries is within the ±1mm tolerance in both X and Y directions for all measurements. A novel procedure to measure the functionality of the first scatterers in the US gantry is included in the QA procedure. It was found to be sensitive enough to pick up the thinnest scatterer of 0.6 mm in both possible failure methods — when it always remains in the beam or in the case when it never goes into the beam. The daily QA procedure presented here can be implemented at PBS or US proton therapy centers with a minimal outlay for equipment and setup time. The procedure can be performed in less than 30 min, and has been found to be accurate and reliable enough for the QA of a proton therapy gantry before patient treatment every day.

PACS number: 87.55.Qr

## I. INTRODUCTION

A thorough quality assurance (QA) program is essential in any radiotherapy modality to ensure the safe and accurate treatment of patients. The limiting factors for the QA program are most often the time required and the measurement equipment that is currently available. Proton therapy is relatively new and the number of centers is small compared to photon therapy, and consequently there is a limited number of dosimetry tools specifically designed for proton therapy QA. There are a number of devices available that are designed to measure a specific property of the proton beam, which together could be used to create a thorough QA program.

Range and SOBP measurements can be accurately done in a single measurement with a stack of scintillating plates, such as the 28×3mm thick plastic scintillators presented by Amaldi et al.^(1)^ or using a Zebra (IBA Dosimetry, Schwarzenbruck, Germany), a commercial multilayer ionization chamber.^(2)^ It is also possible to measure the output with a single measurement using an ionization chamber in a solid phantom, or the collinearity of the proton beam axis and X‐ray beam axis using a film or ionization chamber array. Similarly it is possible to accurately measure the spot position accuracy using a scintillation screen, such as the Lynx 2D (Fimel, Paris, France). But to measure all of these properties on a daily basis before patient treatment is too time‐consuming and not practical if a separate device must first be set up and aligned for each check.

Ding et al.^(3)^ published a method of using a commercially available Sun Nuclear QA3 device (Melbourne, FL) designed for photon/electron linacs for the QA of uniform scanning (US) proton beams. They were able to measure the output, range, and symmetry of the beam in a single measurement. Pencil beam scanning (PBS) proton beams have an added complexity that makes daily QA measurements more difficult. Potential treatment errors can occur if the individual spot positions are not accurate or if there is a large change in the spot size due to changes in the beam optics or scattering material in the beam line.

What we present here is a novel method for using a single device for daily QA in PBS and an extension of the device's use in US. The QA3 device has been adapted to measure not only the range and output, but also the collinearity of the X‐ray system and proton beam, spot position, and spot sigma, and to QA the first scatterers and a number of other imaging and mechanical checks.

## II. MATERIALS AND METHODS

There are a number of publications that together have a thorough list of daily QA checks and tolerances for proton therapy.^3^, ^4^, ^5^, ^6^, ^7^, ^8^ In addition to the safety and interlock checks which are performed following methods in these publications, we have developed a method of using a Sun Nuclear QA3 device to perform all of the remaining QA checks in Table 1.

**Table 1:** QA checks performed using the Sun Nuclear QA3 device.

*Category*	*Procedure*	*Modality*	*Tolerance*
Mechanical	Laser alignment	US, PBS	2 mm
Mechanical	PPS absolute position accuracy	US, PBS	2 mm
Mechanical	PPS correction for gantry angle	US	1 mm
Imaging	PPS correction vector	US, PBS	1 mm
Imaging	Connectivity of OIS and imaging system	US, PBS	Pass/Fail
Imaging	Imaging and alignment system functionality	US, PBS	Pass/Fail
Dosimetry	Output	US, PBS	2%
Dosimetry	Range	US, PBS	1 mm
Dosimetry	Proton beam vs. X‐ray collinearity	US	1 mm
Dosimetry	Spot position vs. X‐ray isocenter	PBS	1.5 mm
Dosimetry	Spot sigma	PBS	15%
Dosimetry	WET of first scatterers	US	0.5 mm

### A. Sun Nuclear QA3 device design

**Figure 1:**
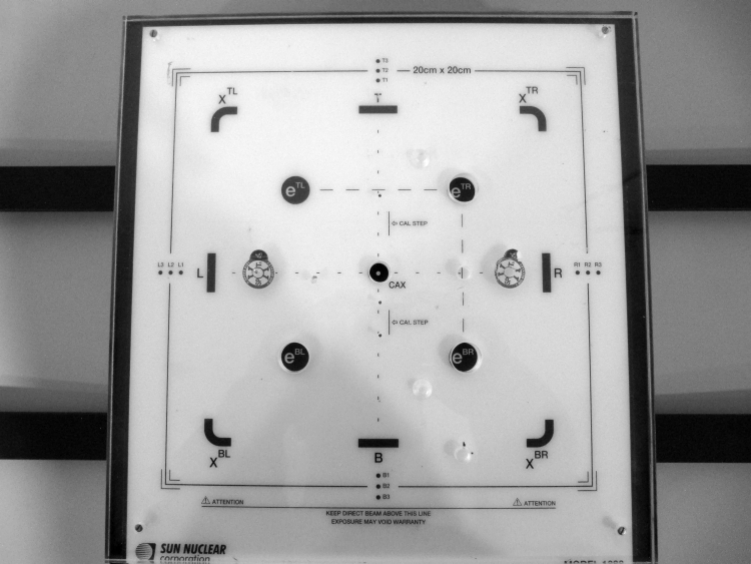
The front of the QA3 device showing the locations and spacing of the ionization chambers and diodes.

The QA3 device is designed for routine QA in photon and electron radiotherapy beams. It contains 12 Precision SunPoint diodes and 13 parallel plate ionization chambers (Fig. 1). The diodes are placed in sets of three along the axis, spaced 5 mm apart from each other, with the center diode 100 mm from the center of the device. The four electron energy chambers have buildup material of air, copper, aluminum, and iron. A Perspex plate with a thickness of $1.1\;g/\;{\text{cm}}^{2}$ is attached to the front of the QA3 device. The plate has radio opaque markers placed along the central axis of the device for alignment. There are holes drilled over three of the electron energy chambers (ETR, E BL, and EBR) and the central chamber. The water‐equivalent buildup in a proton beam of the four electron energy chambers, including the Perspex plate, are: E_TL=41mm, E_TR=2mm, E_BL=7mm, and E_BR=43mm. The central chamber has a buildup of 10 mm and the other eight chambers have 21 mm.

### B. Mechanical and imaging

The daily QA is performed using a QA patient in the oncology information system (OIS) using the same workflow as with real patient treatment. This tests that the connectivity of all of the systems is functional. Problems with sending the correction vectors from the VeriSuite (Medcom, Darmstadt, Germany) imaging and alignment system back to the MOSAIQ OIS (Elekta AB, Stockholm, Sweden) can then be found before a patient is on the table and, in most cases, solved before patient treatment is scheduled to start.

The IBA universal nozzle does not have a fixed isocenter position. Due to a sagging of the components in the nozzle, the central axis of the proton beam does not go through a fixed point in space for all gantry angles. To correct for this, the patient positioning system (PPS) is corrected as a function of gantry angle (in the order of 1.5 mm). This is checked for two gantry angles during the daily QA of the US gantry, which has the universal nozzle.

The QA3 device is attached to a custom‐made Perspex holder containing a set of radiopaque markers, the basic design of which is published in Ding et al.^(3)^ The holder has been improved since the first publication with the addition of a second indexing bar to improve the rigidity of the attachment and, in particular, to reduce rotations. The QA3 device is attached to the patient couch and aligned to the X‐ray isocenter at gantry 0°. Markings on the QA3 device holder have been made to check the alignment of the lasers at isocenter once the device has been aligned. The treatment field, at gantry 90°, is then sent and the PPS position is corrected by the system, as it would be for a patient treatment, and a second set of orthogonal X‐rays is taken. The distance between the markers on the QA3 device and the X‐ray isocenter is measured to verify that the PPS position was adjusted between the two gantry angles correctly. This procedure also checks the functionality of the imaging and alignment system and the accuracy of the correction vector calculation of the VeriSuite system.

The couch coordinates after the alignment are recorded to track the PPS positioning accuracy from day to day. The QA3 device is attached to the PPS using a rigid holder and the same index bars as used in patient treatment. Hence, the PPS position coordinates, when the device is aligned to the isocenter, should be the same from day to day within the positioning tolerance of the system.

### C. Uniform scanning

The output and range checks are similar to those presented by Ding et al.^(3)^ The central chamber is used to measure the output of the proton beam from day to day, and the four electron energy chambers of the QA3 device are used to check the range stability of the beam. During the first weeks of using the QA3 device for daily QA, the dose measurement of the device were cross‐checked with an ionization chamber measurement in a Solid Water phantom using a FC65‐G type Farmer chamber. The QA3 range measurements were also verified by measuring the range with an IBA dosimetry Zebra multilayer ionization chamber (MLIC) over a six‐month period.

The range check has been improved on the method presented by Ding et al.,^(3)^ with the separate software no longer being required to analyze the measurements offline. The QA3 software shows an instant indication if the range is within the given tolerance or not. Four different ranges are checked through the week with a specific compensator made for each of the four options in an IBA universal nozzle. The compensators were designed to have two of the electron energy chambers on the distal falloff of the proton beam, near the 50%, for ranges of 10 cm, 15 cm, 20 cm, and 30 cm. The e‐Energy (electron energy) check in the QA3 software itself can be calibrated so that it will pass if the range is within a given tolerance and fail if it is not. This check is designed to measure changes in electron energy, but can also be used to measure changes in the proton energy (i.e., range). This requires the electron energies in the QA3 software to be calibrated to correspond to different proton ranges. The compensator designed for a 20 cm range proton beam was used for this calibration. A 9 MeV electron energy was calibrated to correspond to a range of 19.8 cm, a 12 MeV electron energy was 20.0 cm, and 15 MeV electron energy was 20.2 cm. The daily QA software template for each of the four proton ranges were designated as a 12 MeV electron energy. Changes in the proton range then result in a change in the e‐Energy check of the QA3 software, which is displayed as an arbitrary percentage value.

The tolerance of the e‐Energy check was set to be equivalent to a proton range error of 1 mm. Although the distal 50% of the proton beam is always located on the same two electron energy chambers, the distal penumbra of the beam increases with increasing range and therefore the tolerance, in percentage of the e‐Energy check, is range‐dependent. The e‐Energy tolerance value for each proton range was determined by measuring with the correct compensator, but changing the range of the proton beam by plus and minus one millimeter.

For the correct alignment of a patient, it is essential that the central axis of the X‐ray system and the central axis of the proton beam are collinear. The angle between the two axes is determined by the physical position of the X‐ray source in the nozzle, which was measured to be acceptable during commissioning. Due to the distance between the source and isocenter, a small shift in the position of the X‐ray source or imaging plane will have a negligible effect on the relative angle of the X‐ray axis to the proton beam axis. To test that the collinearity remains constant, it is therefore adequate to measure the distance between the central axis of the proton beam and the central axis of the X‐ray system at the isocenter plane. We have developed a procedure to measure the collinearity using the QA3 device with the same irradiation as used for the dose and range measurements. The device is aligned to the isocenter using the X‐ray system, as described above. An open 18 cm circular aperture is used with a snout extension of 47 cm; this aligns the edges of the field with the field size diodes on the QA3 device. The readings on the diodes can be used to calculate the shift of the proton beam isocenter from the position of the QA3 device aligned to the X‐ray crosshairs. The accuracy of this method was tested by intentionally shifting the device away from the X‐ray isocenter in both X and Y directions up to 4 mm. Comparisons were also made to film measurements of the collinearity.

During the commissioning of the gantry, we experienced a mechanical failure of one of the first scatters (called lollipops in an IBA gantry). The scattering foil itself came off the support frame, which meant that the switches for the interlocks were still pressed and the system thought that it was moving in and out as expected.

**Figure 2:**
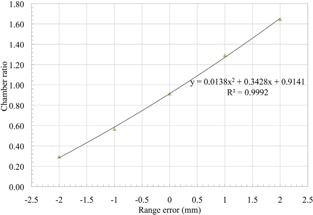
The relationship between the ratio of the e‐Energy chamber readings with all lollipops in and all lollipops out and an introduced range error.

To ensure that this is detected if it happens, we have devised a method that uses the QA3 device to measure the water‐equivalent thickness (WET) of all of the lollipops. The method uses the compensator for the range 20 cm beam daily QA and a second compensator with a WET less by exactly that of the total WET of all of the lollipops. Two measurements are then made, one with all lollipops out with the thicker of the two compensators, and a second measurement with all lollipops in and the thinner compensator. The range of the beam is such that, in both cases, the distal 50% is on two of the e‐Energy chambers, and the other two e‐Energy chambers are on the comparatively flat proximal region of the Bragg peak. The ratio of the distal and proximal chambers readings will be highly dependent on range and can be calibrated to give a measurement of the deviation in range. The difference between chamber ratios of the two readings, one with all lollipops in and the other with all lollipops out and a thicker compensator, can be used to give an error in millimeters in the total WET of the lollipops. This was calibrated by intentionally changing the range of the proton beam by ±2mm for one of the two measurements. The relationship between the ratio of the e‐Energy chamber readings and a deviation in range between the two measurements is shown in Fig. 2. The e‐Energy chamber readings are a ratio between two measurement points on a Bragg peak and are, therefore, independent of the dose delivered. The WET measurement of the lollipops was found to be unaffected, even when the dose delivered for each of the two readings differed by a factor of two.

The thinnest lollipop has a WET of 0.6 mm, which must be detectable by this method in both possible failure methods — where it always remains in the beam and where it always remains out of the beam. This was tested by measuring the second field with all lollipops in except one, and with the first field with all lollipops out except one. The variation in readings due to noise and variations in the beam between the two measurements must also be much less than 0.6 mm so that there are no false‐positives. The two measurements are done immediately after each other without changing the settings in the beam line, so the range of the proton beam for the two measurements during one daily QA is the same. But the accuracy of the procedure must not be affected by small variations in the range of the proton beam from day to day, since the range accuracy of the beam is in the order of 1 mm and the smallest lollipop that must be detected is only 0.6 mm WET. Because the measurement is based on a ratio between two measurements variations in the range output of the gantry should not affect the reading. This effect of small changes in range of the proton beam was tested by intentionally changing the proton beam range for the measurement up to 2 mm in either direction, while still using the same range for both measurements.

### D. Pencil beam scanning

**Figure 3:**
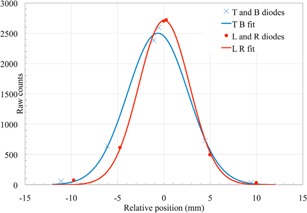
The spot size and position measured with the twelve field size diodes in X (LR diodes) and Y (TB diodes) and a Gaussian fit.

Similar to the collinearity test in US, the alignment between the X‐ray system and the proton beam is measured by aligning the QA3 device to the X‐rays and measuring the position of the proton beam with the four sets of three diodes. The two sets of three diodes in the left–right (LR) direction and similarly the two sets in the top–bottom (TB) direction are located along the axis at nominal distances of 95 mm, 100 mm, and 105 mm from the central of the QA3 device (Fig. 1). A single 220 MeV spot is directed to the innermost diode of each triplet and the remaining two diodes of each triplet sample the outer side of this spot. For the data analysis, we assume that the two spots on each axis are identical. This assumption has been verified with measurements using the Lynx 2D scintillating screen. The measurements from the two sets of diodes on each axis are combined into a single sampling grid of 20 mm width with a 5 mm sampling distance. In the Gaussian fitting, we include an offset in the diode measurement positions to account for the fact that the diodes are not placed at exactly 95 mm, 100 mm, and 105 mm from the center of the QA3 device. A Gaussian function is fitted to the six sample points along each axis. Figure 3 shows an example of the data analysis. A relative shift in the positions of the two spots along an axis will decrease the accuracy of the Gaussian fit used to determine both the spot position and spot size. This was tested by intentionally shifting one of the two spots along an axis by ±1mm and comparing the calculated spot position and spot sigma for a series of 20 measurements.

The fit shown in Fig. 3 gives a sigma of 2.8 mm and 3.3 mm, in LR and TB directions, respectively. The shift in LR direction is 0.0 mm and −0.6mm in TB direction. We analyzed the accuracy of this method and set a tolerance of 1.5 mm for the position and a change in spot size of 15% compared to the baseline value. The tolerance in position matches the specification of the beam delivery system, which has a 1 mm accuracy for the relative spot position and 0.5 mm accuracy for the position of the X‐ray isocenter cross and the beam isocenter. A system has been set up using a 2D scintillating screen to calibrate and test the QA3 spot size and shape measurements.

The accuracy of the procedure was tested by shifting the PPS, with the QA3 device attached, by up to 5 mm and comparing the shift to the error in spot position reported by the QA3 measurements. The stability of the spot position was also tested by taking multiple measurements without moving the QA3 device.

Diodes are prone to radiation damage in proton beams and the sensitivity can decrease with accumulated dose over the life of the diode.^(9)^ There are no specifications given for the diodes in the QA3 device, and any change in sensitivity during the daily QA will be higher for the innermost diode which receives the highest dose. A reduction in sensitivity would result in a reduced peak height of the Gaussian fit and, therefore, a larger calculated spot sigma. The effect of radiation damage during the daily QA on the diodes was tracked by comparing the reading in counts of all 12 diodes over the period of one year to the baseline values.

The analyses of lateral properties and the range are implemented in an Octave script (GNU Octave, version 3.4.3). After finishing an acquisition in the QA3 software, we export the corresponding data file and invoke a batch script which applies the octave analysis on the most recent run in the export file.

There are two independent range checks implemented in our PBS version based on two pairs of the electron energy chambers. A 30mm×30mm field is directed on each chamber. A pair of chambers is irradiated with the same beam energy, but the buildup on the chambers differs: the ‘left’ chamber samples dose in the entrance plateau of the monoenergetic beam, and the ‘right’ chamber in the distal falloff region. The buildup comprises the internal buildup of the QA3 device and a PMMA block which is mounted on both ‘right’ chambers. The two beam energies used are 187 MeV and 201 MeV, which match the total buildup on the two right‐hand chambers to have the distal 50% on the chamber. The ‘left’ chamber serves as a reference chamber and we use a function of the ratio of ‘right’ to ‘left’ to calculate the shift in range. With a similar concept to US, a change in range will result in a change in ratio of the readings of a pair of chambers. The accuracy of the method was tested as a function of range for −2mm to 2 mm around nominal range. This was done by changing beam energy according to ICRU 49 energy‐range relation. We also validated this measurement by putting thin polystyrene sheets as additional buildup. The difference in range as a function of dose ratio ‘left‐to‐right’ was parameterized with a 2nd order polynomial. This inverse function is included in our octave script, and displays shift in range for the ‘bottom’ pair of chambers and for the ‘top’ pair of chambers. The tolerance on the daily QA range measurement is 1 mm.

During commissioning the absolute dose calibration of the treatment planning system for PBS was done by measuring the dose at a fixed depth of 30 mm in water for a monoenergetic proton beam. A 10cm×10cm pristine layer with 2.5 mm spot spacing and 1 MU per spot was measured for proton energies of 100 MeV to 226.7 MeV in 5 MeV steps. The output of the proton beam is energy‐dependent, but this energy dependence will not change from day to day. Variations in the output from day to day will be due to changes in the sensitivity of the monitor chamber in the nozzle (for example, due to the incorrect entering of the temperature and pressure values or leakage in the signal cable). During the daily QA, the dose accuracy is checked by irradiating the center chamber of the QA3 device with a single 30mm×30mm pristine layer of 159 MeV. With a water‐equivalent buildup of 1 g/cm^2^, the chamber measures dose in the flat entrance part of the depth profile, as was done for the absolute dose calibration of the planning system. We use the default QA3 software for the dose analysis to compare the dose measured on the day to the baseline value. The dose measurements were cross‐checked against measurements with an Exradin T1 thimble ionization chamber (Standard Imaging Inc., Middleton, WI) in water.

## III. RESULTS & DISCUSSION

### A. Mechanical and imaging

**Figure 4:**
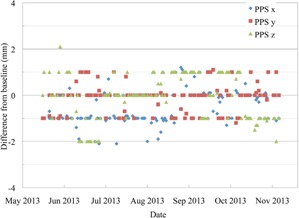
The absolute PPS position after the QA3 device has been aligned to the X‐ray crosshairs in the US gantry, shown as the difference from the baseline position.

After six months of performing daily QA with this procedure, we found that the alignment of the markers on the QA3 were always within tolerance after the correction was made with the VeriSuite X‐ray imaging system. This indicates a stable functionality of the correction vector calculation and transfer. It also shows that the PPS correction for gantry angle for the two angles is within tolerance. Figure 4 shows the difference in the absolute PPS position from the baseline position, after the QA3 device was aligned to the X‐ray crosshairs in the US gantry. These PPS positions remained within ±2.1mm, with the largest variation in the Z direction (height of the couch) and the majority of points within ±1mm of the baseline value. The differences in the PPS position from day to day are due to a combination of four factors: the reproducibility of the alignment of the QA3 device on the couch, the alignment of the device to the X‐ray crosshairs by the user (1 mm tolerance), the precision of the absolute position of the couch itself (IBA specified accuracy is ≤0.5mm for 67% of cases), and the gantry correction file (IBA specified accuracy is ≤0.5mm for 67% of cases).

**Figure 5:**
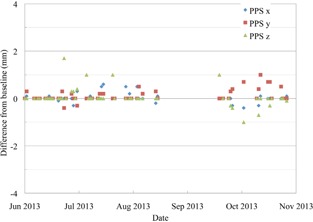
The absolute PPS position after the QA3 device has been aligned to the X‐ray crosshairs in the PBS gantry, shown as the difference from the baseline position.

Figure 5 shows the absolute PPS position after the QA3 alignment in the PBS gantry. All of the points are within the tolerance of 2 mm since the QA was started in June 2013. The PBS gantry has a fixed isocenter position so, unlike the US gantry, there is no PPS correction for gantry angle. Instead the beam itself is steered by the scanning magnets so that the central axis of the beam goes through a fixed point in space for all gantry angles. The requirement to move the PPS in the US gantry to correct for changes in gantry angle contributes to the larger deviations in the PPS position seen.

### B. Uniform scanning

The daily QA of the US gantry requires three irradiations of the QA3 device. The first is for the output, range, and collinearity tests and the last two are specifically to test the WET of the lollipops. The output factor has been measured in the US gantry for a longer period of time than the other tests. The dose readings from the QA3 device agreed with dose measurements using an FC65‐G Farmer chamber to within 1% over the period of two months where both measurements were performed.

**Figure 6:**
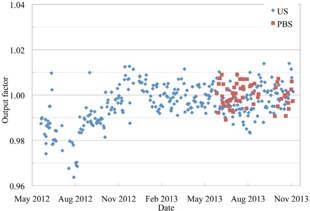
The output factor of the proton beam measured with the central ionization chamber of the QA3 device for both the US and PBS gantries.

During the first six months of running the daily QA procedure, the output factor was found to drift by about 2% (Fig. 6). The same trend was seen in the FC65‐G chamber readings during the time when both measurements were performed daily. This happened during the commissioning of the gantry and there were a number of possible causes suggested. Two of the possible causes were an inadequate regulation of the temperature in the treatment room and gantry pit or a problem with the ionization chamber in the nozzle. It is a slow trend with as much variation from day to day as from one month to the next, and a number of fixes were made to correct it. Since around January 2013 for US, and since the PBS daily QA started in June 2013, the output has remained within the 2% tolerance, with the majority of measurements within ±1%.

**Figure 7:**
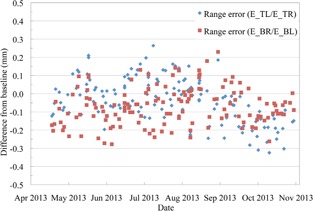
The range error in the US gantry measured using four ionization chambers. ETL and EBR are near the distal 50% and ETR and EBL are on the flat plateau region of the SOBP.

The range of the US beam remained within ±0.5mm during the entire six months that it was measured with the QA3 device (Fig. 7). The results are shown as the deviation from the baseline measurement of a 10 cm, 15 cm, 20 cm, and 30 cm range beam. The range 20 cm beam is measured twice a week and the other three ranges are measured once per week. The range measured with the MLIC remained within 0.5 mm of the baseline values, with most readings within 0.1 mm over the six‐month period.

**Figure 8:**
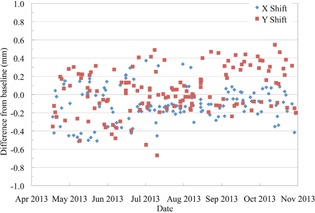
The centering of the US proton beam on the QA3 device which has been aligned to the X‐ray crosshairs.

The centering of the proton beam on the QA3 device during the daily QA, calculated using the twelve diodes is shown in Fig. 8. There is an offset between the baseline reading and the center of the QA3 device due to the positioning of the radiopaque markers relative to the diodes. Therefore, the results are shown as the shift in X and Y relative to the baseline, with the measured shift in both X and Y all within the 1 mm tolerance. The absolute alignment between the proton beam and X‐ray system is checked in the monthly QA with EBT3 film, the daily QA procedure ensures that there is no change during the month in the proton beam and X‐ray system collinearity. The position offset calculated with the QA3 device agreed with the actual shift when the PPS was intentionally moved in 1 mm steps from −4mm to +4mm, to within 0.5 mm when the shift was along a single axis and within 0.7 mm when there was a shift in both X and Y directions.

The lollipop QA procedure detected an error in the total WET of the lollipops of 0.6±0.1mm when the thinnest lollipop of 0.6 mm WET always remains in the beam or in the case when it never goes into the beam. Based on this measurement, a tolerance of 0.5 mm was set for this QA test to detect a malfunction of any of the lollipops. The procedure is also robust against any changes in the proton beam range from day to day. The measured total WET was within 0.1 mm for any incident proton beam range between 19.8 cm to 20.2 cm.

**Figure 9:**
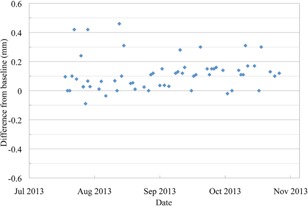
The difference in the measured total WET of the lollipops from the baseline measurement.

Since the test started in the middle of July 2013, all of the measurements are within the 0.5 mm tolerance (Fig. 9). The average of the readings differs from the baseline by 0.1 mm. This has now been corrected to reduce the chances of a false‐positive or missing the case when the smallest lollipop does not move into the beam, which would result in a −0.6mm WET measurement.

### C. Proton beam scanning

**Figure 10:**
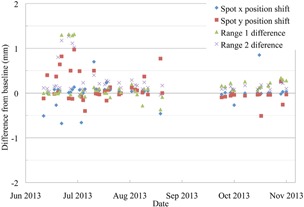
The range accuracy of a 187 MeV and a 201 MeV PBS proton beam, and the spot position accuracy of a 220MeV beam.

The daily QA of the PBS gantry is performed with a single irradiation of the QA3 device. The output of the PBS beam, as shown in Fig. 6, has remained within the 2% tolerance and almost all measurements are within 1% of the baseline. The stability of the range of two single energy 30×30mm scanned layers is shown in Fig. 10. There is a set of four measurements within the first month that have a shift of about 1 mm from the other measurements. This was due to the incorrect orientation of an almost square cross‐section buildup block placed over two of the chambers on the QA3 device. The procedure was updated to prevent this from occurring and the range of both energy layers has remained within ±1mm and, after the new baseline in October, the range has remained within ±0.5mm. The spot position is within 1 mm of the baseline position for all QA measurements since the procedure was implemented. The apparent increase in the accuracy after the October baseline is due to improvements in the fitting of the data used to calculate the deviation and not from any changes in the proton beam itself. The spot position error measured with the QA3 agreed with actual shift to within 0.5 mm when the PPS was shifted in a single direction from 1 mm to 3 mm. When the PPS was shifted by 5 mm along a single axis, the QA3 device reported the shift along that axis to within 0.8 mm, but also incorrectly showed a shift along the other axis of up to 2.5 mm. An error in spot position as large as 5 mm is not expected, but, in the case where it would happen, the QA3 device will overestimate the error and therefore still inform the user that there is a problem in the QA measurement.

**Figure 11:**
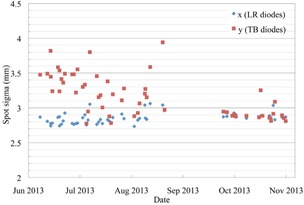
The spot sigma of a single 220 MeV proton beam spot, shown in the X and Y directions, measured by the 12 diodes on the QA3 device.

Figure 11 shows the spot sigma from the Gaussian fit of the diode measurements of 220 MeV spots. The sigma in the Y direction was initially erratic with deviations outside the tolerance of ±15%. The measurements performed with a 2D scintillation screen do not show the same erratic behavior. The cause was found to be the alignment of the spots on the diodes due to an offset between the radiopaque markers on the QA3 holder and the center of the QA3 device. When the QA3 device was aligned to isocenter using the radiopaque markers, the device was shifted too far in one direction, which resulted in an inaccurate calculation of the spot sigma. The alignment of the device in the Y direction was fine‐tuned by moving the markers, and the data used for the Gaussian fitting algorithm were updated. The measured spot sigma after these improvements at the start of September is much more stable and matches the actual spot sigma, as measured with a 2D scintillating screen, more closely.

By intentionally shifting one of the two spots along an axis by ±1mm, the calculated spot position changed by 0.5 mm, and the spot sigma changed by −0.28mm when the two spots moved closer together and +0.27mm when they moved further apart. The reported spot position correctly shows the average of the two spot positions, but the calculation of the spot sigma changes by approximately 9% for a 1 mm relative shift of the two spots.

The change in response of the individual diodes was analyzed and found to remain constant over a period of one year. The innermost diodes, which receive the highest dose, had no reduction in the average number of counts for the single irradiation during the daily QA. These diodes are only irradiated by the entrance dose of a single spot each day, and over the period of a year the total dose was not high enough to show any measureable damage.

## IV. CONCLUSIONS

The daily QA procedure presented here can be implemented at other proton therapy centers which have pencil beam scanning or uniform scanning only, or in centers like ours with multiple modalities. Using this procedure, it is possible to measure both the spot position accuracy and sigma in a PBS beam, as well as the range accuracy and output of any proton therapy beam, all in a single irradiation. The procedure can be performed in less than 30 min, and also checks the imaging and alignment system, collinearity, functionality of the first scatterers, and the absolute PPS position — all with a single device. The procedure was found to have a high enough accuracy to test if all of these properties are within their tolerances. During our experience of performing the procedure every day before patient treatment, we have found it to be accurate and reliable, and the results show that the proton therapy system is stable and remains within the tolerances given.

## References

[1] Amaldi U , Hajdas W , Iliescu S , et al. Advanced quality assurance for CNAO. Nucl Instrum Meth A. 2010;617(1–3):248–49.

[2] Dhanesar S , Sahoo N , Kerr M , et al. Quality assurance of proton beams using a multilayer ionization chamber system. Med Phys. 2013;40(9):092102. 24007171 10.1118/1.4817481

[3] Ding X , Zheng Y , Zeidan O , et al. A novel daily QA system for proton therapy. J Appl Clin Med Phys. 2013;14(2):115–26. 23470936 10.1120/jacmp.v14i2.4058 PMC5714372

[4] Arjomandy B , Sahoo N , Zhu XR , et al. An overview of the comprehensive proton therapy machine quality assurance procedures implemented at The University of Texas M. D. Anderson Cancer Center Proton Therapy Center‐Houston. Med Phys. 2009;36(6):2269–82. 19610316 10.1118/1.3120288

[5] ICRU . Prescribing, recording, and reporting proton‐beam therapy. ICRU Report No. 78. Oxford, UK; Oxford University Press; 2007.

[6] Kase Y , Yamashita H , Numano M , Fuji H , Murayama S . A revision of proton machine quality assurance for wobbled‐proton‐beam therapy. Radiol Phys Technol. 2013;6(2):444–52. 23689901 10.1007/s12194-013-0217-2

[7] Klein E , Hanley J , Bayouth J , et al. Task Group 142 report: quality assurance of medical accelerators. Med Phys. 2009;36(9):4197–212. 19810494 10.1118/1.3190392

[8] Kutcher GJ , Coia L , Gillin M , et al. Comprehensive QA for radiation oncology: report of AAPM Radiation Therapy Committee Task Group 40. Med Phys. 1994;21(4):581–618. 8058027 10.1118/1.597316

[9] Pacilio M , De Angelis C , Onori S , et al. Characteristics of silicon and diamond detectors in a 60 MeV proton beam. Phys Med Biol. 2002;47(8):N107–N112. 12030565 10.1088/0031-9155/47/8/403

